# Future Therapy for Hepatitis B Virus: Role of Immunomodulators

**DOI:** 10.1007/s11901-016-0315-9

**Published:** 2016-11-10

**Authors:** Edward A. Pham, Ryan B. Perumpail, Benjamin J. Fram, Jeffrey S. Glenn, Aijaz Ahmed, Robert G. Gish

**Affiliations:** 1 Department of Medicine, Division of Gastroenterology and Hepatology, Stanford University School of Medicine, Stanford, CA USA; 2 Department of Microbiology and Immunology, Stanford University School of Medicine, Stanford, CA USA; 3 Veterans Administration Medical Center, Palo Alto, CA USA; 4 Hepatitis B Foundation, Doylestown, PA USA

**Keywords:** Hepatitis B, Immune modulators, Toll-like receptor agonists, Checkpoint inhibitors, Therapeutic vaccines, Engineered T cells, RNA interference, CRISPR/Cas9

## Abstract

Although currently available therapies for chronic hepatitis B virus infection can suppress viremia and provide long-term benefits for patients, they do not lead to a functional cure for most patients. Advances in our understanding of the virus-host interaction and the recent remarkable success of immunotherapy in cancer offer new and promising strategies for developing immune modulators that may become important components of a total therapeutic approach to hepatitis B, some of which are now in clinical development. Among the immunomodulatory agents currently being investigated to combat chronic HBV are toll-like receptor agonists, immune checkpoint inhibitors, therapeutic vaccines, and engineered T cells. The efficacy of some immune modulatory therapies is compromised by high viral antigen levels. Cutting edge strategies, including RNA interference and CRISPR/Cas9, are now being studied that may ultimately be shown to have the capacity to lower viral antigen levels sufficiently to substantially increase the efficacy of these agents. The current advances in therapies for chronic hepatitis B are leading us toward the possibility of a functional cure.

## Introduction

With an estimated 240 million people worldwide chronically infected with hepatitis B virus (HBV), it is among the leading causes of liver disease, cirrhosis, and hepatocellular carcinoma [1]. Current treatments for HBV can suppress viral replication but rarely result in a cure and often require prolonged and possibly lifelong administration. Newer and more effective therapies are needed to achieve sustained and durable remission and potentially even a functional cure for the majority of patients.

## Brief Overview of HBV Virology

HBV belongs to the Hepadnaviridae family. The HBV virions, also known as Dane particles, contain a lipid-derived viral envelope with embedded viral surface proteins (hepatitis B surface antigen/HBsAg) and an interior nucleocapsid protein (hepatitis B core antigen/HBcAg) that forms an icosahedral shell which harbors a partially double-stranded, relaxed circular DNA genome [2]. Upon infection of target hepatocytes, the viral genome is released into the cytoplasm, transported to the nucleus, and then converted to a minichromosome known as the covalently closed circular DNA (cccDNA). This cccDNA acts as a template for multiple viral mRNA transcripts synthesized via host RNA polymerase II. However, only the pre-genomic RNA (pgRNA) is incorporated into the nucleocapsid together with the HBV polymerase. After viral pgRNA encapsidation, a somewhat complex replication scheme involving reverse transcription generates the relaxed circular DNA, and the genomic RNA is degraded. The unusual stability of the cccDNA due to folding, episomal changes, and modifications is thought to be one major reason for the persistence of HBV. The other reason for persistence is that HBV has the ability to train and evade the host immune responses which ultimately are required for viral control, viral clearance, and possibly a cure. People who recover from acute HBV infection typically achieve HBsAg loss, with antibody to HBsAg (anti-HBs) seroconversion indicating a high level of immune control. Even these patients, however, harbor cccDNA and pre-genomic viral transcripts in hepatocyte nuclei for life [2, 3], potentially leading to reactivation when such patients undergo chemotherapy or other immunosuppressive therapies [4] or when those with hepatitis C virus (HCV) coinfection are treated with HCV direct acting antiviral agents [5]. Thus, most experts now agree that while an absolute “sterilizing” cure of HBV that eliminates all forms of HBV replication intermediates including cccDNA will be very difficult to achieve, a functional cure in which patients achieve sustained absence of HBV viremia and loss of HBsAg after a defined course of therapy and are returned to a state of health equivalent to that of a person who spontaneously recovers from acute HBV infection may be an achievable goal with the next wave of new HBV therapies. Immunomodulating therapies, the topic of discussion in this review, are likely to be an important component of these new therapeutic options.

## Current Therapies

Current first-line therapies for chronic hepatitis B remain limited to pegylated interferon-alpha (PEG-IFN-α) and nucleos(t)ide analogues (NAs). NAs inhibit the reverse transcriptase activity of the HBV polymerase and lead to a rapid decrease in HBV viremia [6]. These agents, however, have little if any effect on the immune system and offer at best slight reductions of cccDNA and thus result in a very low rate of HBeAg and HBsAg loss and rarely achieve anti-HBe and anti-HBs seroconversion [6]. The other first-line therapy, the immune modulatory agent IFN-α, or the pegylated version PEG-IFN-α, belongs to a class of antiviral cytokines which are proteins naturally secreted by the host during viral infections. Despite its discovery almost 60 years ago [7], the molecular mechanisms by which IFN-α exerts its antiviral effects are still poorly defined given its pleiotropic effects that result in stimulation of both the innate and adaptive immune systems. Compared to the nucleos(t)ide analogues, treatment with PEG-IFN-α results in slower clearance of HBV viremia but a higher, although still modest, rate of HBeAg and HBsAg loss and anti-HBe and anti-HBs seroconversion [8]. Lengthier treatment with IFN has been shown to achieve higher rates of HBsAg loss. In a small study of 10 HBV patients who were HBsAg-positive and HBeAg-negative with fully suppressed HBV DNA for at least 3 years, the addition of peg-IFN to standard treatment with nucleos(t)ide analogues led to a loss of HBsAg in 40 % of patients at 48 weeks and in 60 % of patients at 96 weeks with no relapse during 12–18 months of follow-up; HBsAg to anti-HBs seroconversion was observed in two patients [9•].

IFN-α has been shown to induce epigenetic changes in the cccDNA resulting in transcriptional repression [10]. It is also known to upregulate APOBEC3A, a member of the APOBEC3 family of DNA editing enzymes that deaminate foreign double-stranded DNA cytidines to uridines and target the foreign DNA for degradation [10, 11]. This combination of direct intracellular antiviral activity and stimulation of antiviral immune cells is likely responsible for the higher rate of functional cure in HBV patients treated with IFN. In fact, PEG-IFN-α is the only finite therapy for HBV, but it requires parenteral delivery and is associated with significant adverse effects in many patients, rendering it impractical to administer to all HBV patients. In addition, long-term exposure to systemic IFN has recently been shown to impair the antiviral efficacy of the immune system [12]. However, the higher rate of functional cure obtained with PEG-IFN compared to NAs suggests that to achieve HBV functional cure, future therapies that seek to restore optimal host immune responses to HBV may have the best chance of success.

It has recently been suggested that nucleos(t)ide analogues can also have weak direct immunomodulatory effects, each with a different profile. For example, tenofovir can increase the production of multiple cytokines, including increasing TNF-α and IL-6 in monocytes, and enhancing IL-12 production while decreasing IL-10 expression in human PBMC [13, 14]. In the woodchuck model of HBV, it was shown that entecavir, but not lamivudine, can enhance the effects of therapeutic vaccination [15, 16]. Since newer therapeutic strategies are likely to be used in combination with existing NAs, it is also important to understand the immunological profile exerted by these NAs.

Thymosin alpha 1 (Tα1) is a 28-amino acid peptide that is known to enhance T cell, dendritic cell, and antibody responses and to modulate cytokine and chemokine production [17, 18]. Currently in late stage clinical development in the USA and Europe, it is already approved in 35 countries for treatment of chronic hepatitis B and/or chronic hepatitis C. Because it enhances responses to antigens and T cell maturation, it has been thought that it might help mount a defense against chronic HBV infection [19]. It has been studied both as a sole therapy in chronic HBV patients and in combination with PEG-IFN or NAs. Although early small studies suggested that it might be an effective treatment when used alone, a larger multicenter trial did not confirm its efficacy as a sole treatment [20]. A meta-analysis of 8 trials (583 patients) that compared the effect of lamivudine monotherapy with that of combined lamivudine and Tα1 found that the combination treatment resulted in significantly better ALT normalization rate (80.2 vs 68.8 %, *P* = 0.01), virological response rate (84.7 vs 74.9 %, *P* = 0.002), and HBeAg seroconversion rate (45.1 vs 15.2 %, *P* < 0.00001). A meta-analysis of 7 trials (535 patients) comparing IFN monotherapy to the combination of IFN with Tα1 found that the combination resulted in significantly better results, both at the end of the treatment and the follow-up, in HBV DNA negative rate (54.9 vs 36.3 % at end of treatment, *P* < 0.01; 58.6 vs 30.7 % at follow-up, *P* < 0.01), ALT normalization rate (74.5 vs 60.9 %, *P* < 0.01; and 74.0 vs 55.6 %, *P* < 0.01), HBeAg loss rate (56.9 vs 36.7 %, *P* < 0.01; and 62.2 vs 33.2 %, *P* < 0.01), and HBeAg seroconversion rate (40.1 vs 29.0 %, *P* < 0.05; and 47.0 vs 29.5 %, *P* < 0.01), as well as in HBsAg loss only at the end of the follow-up (9.8 vs 3.7 %, *P* < 0.05) [21]. There are ongoing trials looking at various combinations of Tα1 with other therapies, including the combination of PEG-IFN with Tα1 and of adefovir with PEG-Tα1.

The subject of nucleos(t)ide analogues and other direct acting antivirals (DAAs), IFN, and thymosin alpha-1 in HBV has been discussed in other reviews [22–24]. In this review, we will focus on recently developed immunomodulatory agents that are being tested for treatment of patients with chronic HBV (Table 1).

**Table 1:** Immunomodulatory agents in studies for treatment of chronic HBV agent type modality/stage sponsor compound type and results to date

ABX203	Therapeutic vaccine	Recombinant HBsAg/HBcAg	Phases 2 and 3 [45]	Abivax S.A.
Birinapant	SMAC mimetic/IAP antagonist	Cellular inhibitor of apoptosis proteins	Pre-clinical [30]	TetraLogic Pharmaceuticals
CYT107	Cytokine	Interleukin-17	Phases 1 and 2 [52]	Cytheris S.A.
GS-4774	Therapeutic vaccine	Recombinant inactivated yeast cells expressing HBsAg, HBcAg, HBx	Phase 2 [43 44•]	Gilead Sciences
GS-9620	Pattern recognition receptor	Small molecule TLR7 agonist	Phase 2 [27, 28 29•]	Gilead Sciences
INO-1800	Therapeutic vaccine	Multi-antigen DNA immunotherapy targeting HBsAg and HBcAg	Phase 1 [11]	Inovio Pharmaceuticals with Hoffman-La Roche
SB-9200	Viral sensor activator and cytokine inducer	Small molecule nucleic acid hybrid that activates RIG-I and NOD2 and induces interferon signaling pathways	Pre-clinical [30]	Spring Bank Pharmaceuticals
TG1050	Immuno-therapeutic	Non-replicative adenovirus encoding fusion protein (truncated HBV core, modified HBV polymerase, 2 HBV envelope domains)	Phase 1 [46]	Transgene
Thymosin alpha-1	Biological response modifier	28-Amino acid fragment that promotes differentiation of T cells to a mature stage	Approved in 35 countries; late stage clinical development in the USA and Europe [17–21]	Multiple companies

*Abbreviations*: *HBcAg* hepatitis B core antigen, *HBsAg* hepatitis B surface antigen, *HBx* hepatitis B X protein, *IAP* inhibitor of apoptosis protein, *NOD2* nucleotide-binding oligomerization domain-containing protein 2, *RIG-I* retinoic acid-inducible gene I, *SMAC* second mitochondrial-derived activator of caspases, *TLR7* toll-like receptor 7

## Toll-Like Receptor Agonists

Toll-like receptors (TLRs) belong to a class of receptors known as pattern recognition receptors (PRRs), an important component of innate immune defenses. These receptors sense the presence of foreign pathogens and trigger the release of inflammatory cytokines to induce an antimicrobial state and facilitate subsequent adaptive immune responses. Agonists of TLRs, including of TLRs 3, 8, 7, and 9, have been shown to have anti-HBV effects in animal models [25]. Of these, GS-9620, an orally available TLR7 agonist, is the most advanced in development. TLR7 is a PRR that is mainly expressed in plasmacytoid dendritic cells (pDCs) and B cells. Upon stimulation of TLR7, pDCs produce high levels of IFN and other cytokines, resulting in activation of natural killer cells and cytotoxic T lymphocytes [26].

In chimpanzees, after 8 weeks of GS-9620 treatment, HBV viral load was reduced by more than 2 logs with a greater than 50 % reduction in serum HBsAg and HBeAg [27]. In woodchucks, GS-9620 treatment for 4–8 weeks resulted in a greater than 6 log viral load reduction, loss of HBsAg and seroconversion in a subset, and a reduction of hepatocellular carcinoma (HCC) incidence from 71 % in the placebo group to 8 % in the treated group [28]. In 2 phase 1b trials in which GS-9620 was given as a single dose or as two doses 7 days apart, GS-9620 induced peripheral interferon-stimulated gene 15 (ISG15) expression but had no effect on HBsAg levels or HBV DNA [29•]. Ongoing phase 2 trials of longer duration are assessing GS-9620 in combination with tenofovir. GS-9620’s advantages include oral availability compared to parenteral delivery for PEG-IFN-α and its ability to induce IFN intrahepatically without systemic induction of IFN. By locally inducing the IFN response at the infected site, it is possible that GS-9620 might leverage the beneficial effect of IFN, while avoiding the adverse effects and thus increase patient adherence. However, based on the research to date, it appears that there is a low probability of the successful use of TLR agonists with CHB as monotherapy. If they are to be used, it will almost certainly have to be in combination with antivirals.

In addition to TLR agonists, many other small molecule modulators of innate immunity are being tested for their ability to clear HBV: SB-9200, which activates the RIG-I/NOD2 pathway, and birinapant (TL32711), which is a second mitochondrial-derived activator of caspases (SMAC) mimetic that antagonizes cellular inhibitor of apoptosis proteins (cIAPs) to improve TNF-mediated killing of HBV-infected cells [30].

## Immune Checkpoint Inhibitors

The phenomenon of immune exhaustion was first identified in chronic lymphocytic choriomeningitis virus (LMCV) in mice and was later found to occur in other human chronic viral infections such as HIV, HCV, and HBV, as well as in various cancers. A hallmark of T cell exhaustion in both such viral infections and cancer is the increased expression of various inhibitory receptors such as programmed death-1 (PD-1), cytotoxic T-lymphocyte antigen-4 (CTLA-4), cluster of differentiation 244 (CD244), cluster of differentiation 160 (CD160), and others [31]. In cancer immunotherapy, the use of checkpoint inhibitors such as those that block the PD-1:PD-L1 pathway has resulted in significant clinical benefits with a wide range of cancer types including melanoma, non-small cell lung cancer (NSCLC), and renal cell carcinoma (RCC) [32]. The fact that T cell exhaustion is a major factor in allowing both the progression of these cancers and the persistence of chronic viral infections like HBV suggests that checkpoint inhibitors may potentially achieve clinical benefits when used as treatments for chronic HBV. In a study that used liver biopsies from 42 patients with chronic HBV infection, it was shown that when T cells were incubated with HBV peptides in the presence of anti-PD-L1, blocking the PD-1:PD-L1 interaction led to increased intrahepatic CD8 T cell proliferation and production of IFN-gamma and IL-2 [33].

In a proof-of-concept evaluation of nivolumab, an anti-PD-1 monoclonal antibody, in chronic HCV patients, 15 % of patients had a significant reduction in HCV RNA following a single dose [34], suggesting that checkpoint inhibitors may have antiviral efficacy that might also be effective with chronic HBV. With many more agents targeting the PD1:PD-L1 pathway now in advanced clinical development and some already approved for treatment of several cancers, further testing of these agents’ antiviral efficacy will reveal their potential for treatment of chronic HBV. Based on the research done to date, it appears that there is a moderate to high probability that checkpoint inhibitors could be at least a component of effective therapy for chronic HBV.

However, the risk profile with this therapeutic class is substantial, and caution will have to be taken when considering the use of these agents. There is a considerable risk of adverse effects with any agent in this class, including an increased risk of viral infections, autoimmune reactions or exacerbation of autoimmune disease and, in the immune compromised, opportunistic infections. A recent assessment of T cell transcriptomes confirmed that an exhausted profile is seen in patients with poor outcome in chronic infection, while it correlates with favorable prognosis in autoimmune disease [35]. Therefore, the use of checkpoint inhibitors in chronic HBV infection will have to balance the possible clinical benefits and the risks related to the use of these therapies and will require close and careful monitoring. Given that most of the safety and efficacy profiles of these agents were established in oncology patients, the extent to which those parameters are appropriate for HBV patients without concomitant cancer remains to be determined. Recent phase 1/2 evaluations of nivolumab in HCC patients, 25 % of whom were chronic HBV patients, showed a 19 % objective response rate [36], suggesting that in the field of HBV, therapeutic blockade of PD1:PD-L1 will likely be employed first in those with HCC.

## Therapeutic Vaccines

Therapeutic vaccines are being intensively researched as possible immunotherapies for both chronic viral infections and cancer. In the case of chronic HBV, the goal of therapeutic vaccination is to restore the diminished T cell response to HBV antigen in a robust and specific manner in order to achieve long-term viral control and minimize host cell damage. Strategies for therapeutic vaccines include the HBsAg-based vaccine [37], those that are based on the HLA-A2 restricted HBV peptide to induce specific CD8+ T cell responses [38] and those that are based on a DNA vector encoding various HBV genes [39, 40]. Although these strategies all demonstrated good safety and tolerability profiles and resulted in induced immunogenicity, clinical trials have yet to demonstrate clinical benefits. One likely explanation for these agents’ lack of efficacy in HBV patients, despite inducing robust immune responses in healthy people, is that for them to have benefit, the exhaustion of both CD4+ and CD8+ T cells in HBV patients that is thought to be caused by prolonged exposure to high levels of soluble HBV antigens (particularly, HBsAg) [41] needs to be reversed, at least partially.

Because high viral antigen levels are known to decrease the effectiveness of therapeutic vaccines [42], reducing the level of viral antigens with the use of nucleos(t)ide analogues or newer therapies might increase the efficacy of therapeutic vaccines. In HCV patients, induction of T-cell immune responses is markedly attenuated when therapeutic vaccines were administered to people with a high level of viremia [42]. In the case of chronic HBV, even with optimal suppression of viremia, the abnormally high level of HBsAg and HBeAg in patients with wild-type infection may further complicate and modulate the efficacy of therapeutic vaccines.

Further optimization of the design of studies of these therapeutic vaccines is ongoing, with many in pre-clinical and clinical development. For example, GS-4774 is derived from a recombinant, heat-killed yeast that encodes a fusion protein of HBsAg, HBcAg, and hepatitis B X protein (HBx). Although GS-4774 was shown to be safe and well tolerated [43], its combination with HBV oral antivirals in 178 non-cirrhotic chronic HBV patients did not result in a significant difference in mean HBsAg levels at either 24 or 48 weeks; 3 patients in the highest dose group had a >0.5 log drop of HBsAg; more patients in the GS-4774 groups had >30 % reductions in HBsAg at week 48; 16 % (5/32) experienced HBeAg loss; and 4 patients had HBeAg seroconversion which did not occur in any patient in the oral antiviral sole treatment group, suggesting that there may be real biological activity [44•]. It is possible that lengthier treatment with immunomodulatory agents may be required to achieve meaningful clinical benefits, as was seen in the use of IFN in the study already discussed in which loss of HBsAg increased from 40 % at 48 weeks to 60 % at 96 weeks. GS-4774 was well tolerated in all patients, suggesting that extending the duration of administration may be a practical strategy that is worth further exploration. Other therapeutic vaccines in development include ABX-203 [45], INO-1800 [11], and TG-1050 [46]. Based on the research to date, it would appear that there is a low to moderate probability of successful use of these agents for treatment of chronic HBV. If they are to be used successfully, it will almost certainly be as part of double or triple combination therapy or sequential treatment.

## Engineered T cells

Another approach to immunotherapy is to engineer patients’ immune cells, such as T cells, to eliminate HBV-infected hepatocytes. This technique, called adoptive cell transfer (ACT), has been employed with early promising results in human cancer therapy [47] including those with HBV-associated HCC [48]. With this approach, T cells are removed from a patient, modified to express receptors specific to the particular type of cancer so that the cells are able to recognize the cancer cells, and then reintroduced into the patient. Holding the most promise for chronic HBV treatment is adoptive transfer of T cells expressing chimeric antigen receptors (CARs) which allow the engineered T cells to recognize specific antigens on target cells [49]. This approach was recently shown to have potential promise in mouse models of HBV [50]. However, there is no human data to show whether this approach can be both successfully and safely used in chronic HBV patients. One of the challenges of this approach is the diversity of antigenic presentation of HBV-infected cells since unlike tumor, which often involves clonal expansion, HBV-infected hepatocytes are likely polyclonal. In addition, this is a highly individualized therapy that is very costly, making it a therapy that is unlikely to be successfully used in the developing world. For now, despite the fact that this approach is considered innovative and exciting, it would seem that there is a low probability that this will become a widely used successful therapy for chronic HBV.

## Other Immune Modulators

Many other modulators of immunity are being tested for their ability to clear HBV. SB-9200, now in pre-clinical testing, is a small molecule nucleic acid hybrid that activates RIG-I and NOD2 and induces interferon signaling pathways [30]. Birinapant (TL32711), also in pre-clinical testing, is a second mitochondrial-derived activator of caspases (SMAC) mimetic that antagonizes cellular inhibitor of apoptosis proteins (cIAPs) to improve TNF-mediated killing of HBV-infected cells [30]. In addition, therapeutic cytokines are being studied as a way to restore T cell homeostasis which is disrupted in chronic HBV infection. Interleukin-7 (IL-7) is needed for the proper development of T cells, B cells, and some dendritic cell subsets. It has been shown that in dendritic cells, IL-7-mediated signaling regulates peripheral CD4+ T cell homeostasis [51]. With pre-clinical data derived from several different models showing that IL-7 has significant immune restoration effects and vaccine adjuvant effects, CYT107 is a second-generation recombinant human IL-7 now being tested in CHB patients in combination with antiviral therapy and vaccine in phase 1 and 2 trials [52, 53].

## Lowering Viral Antigen to Improve Immune Modulator Efficacy

There are currently several cutting edge strategies being studied that may lower viral antigen levels sufficiently to substantially increase the efficacy of some of the immune modulator therapies discussed here, including therapeutic vaccines, IFN, and checkpoint inhibitors, the efficacy of which has been shown to be compromised by high antigen levels [42, 54, 55]. Thus, these strategies might become an important component of a combined approach.

Given that one of the major challenges in achieving functional cure in HBV is the persistence of the mini-chromosomal cccDNA that serves as a template for de novo viral antigen synthesis, a therapeutic strategy that has generated interest is the CRISPR/Cas9 genome-editing tool. CRISPR (clustered, regularly interspaced, short palindromic repeat) is a series of short repeated DNA sequences in the bacterial genome that are flanked by sequences of bacteriophages—viruses that infect bacteria—DNA. It has been shown that the CRISPR/Cas9 system is a way that bacteria develop adaptive immunity against bacteriophages. The CRISPR locus is adjacent to the Cas gene which is a type of nuclease that can degrade DNA. Following the first demonstration that the CRISPR/Cas9 system can be engineered to precisely cut genomic DNA at various sites [56], additional research has shown that this system is an extremely efficient genome-editing tool in a broad range of animals [57]. Unlike previous genome-editing techniques, the CRISPR/Cas9 system is easy to use, less expensive, highly adaptable, and able to offer precise control over genome editing. However, delivery system optimization and clarification will be required for efficient delivery and minimizing of off-target effects. The use of this system to combat chronic human viral infection, although still limited to the laboratory, is predicted to hold significant clinical potential given the pace of discovery and development [57]. Encouragingly, multiple studies have demonstrated the potential of this approach to remove HBV cccDNA, first in cell cultures [58, 59] and more recently in HBV-infected mice [60–62]. Thus, the use of CRISPR/cas9 could be a strategy that would directly remove cccDNA which in turn could lower HBsAg levels. Current efforts are under way to develop a method for safe and efficient delivery of the CRISPR/Cas9. Although this approach is perhaps one of the most scientifically exciting, both theoretical and practical considerations combine to push the reality of such anti-HBV therapies into the future.

RNA interference (RNAi) could also improve the efficacy of some of the immune modulators by lowering HBsAg via elimination of HBV RNA transcripts [63]. In a study that assessed the depth and duration of HBsAg reduction in response to the combination of entecavir and ARC-520, the RNAi therapy that is currently the farthest along in development, it was shown that a substantial and prolonged reduction in viral antigens including HBsAg can be achieved in chronic HBV patients through an RNAi mechanism. This type of substantial reduction could be expected to improve the response to a number of the immune modulators. Because of its design, it is thought that ARC-520 will only require administration once monthly or less, potentially making it a practical component of a combination approach. Safety considerations associated with delivery formulations, and the need to transfect every cell in the liver are practical considerations that will need to be addressed with RNAi therapies.

## Conclusions

The past two decades have witnessed substantial progress in the treatment of chronic HBV, including the introduction of nucleos(t)ide analogues which revolutionized hepatitis B management, allowing for potent suppression of HBV viremia. However, these agents have many limitations, including the need for likely lifelong treatment and the lack of functional cure which is an important goal in order to reduce the burden of HBV-associated cirrhosis and HCC. Recent advances in immunotherapy and its remarkable success in the field of oncology offer new hope that such strategies may hold promise for achieving a functional cure of HBV. Some of these agents are now in advanced clinical development. The use of new strategies like CRISPR/Cas9 and RNAi may increase our ability to lower HBsAg levels and improve the efficacy of several immune modulation therapies. In the foreseeable future, effective approaches to chronic HBV infection may require some combination of ongoing suppression of viral replication, immune checkpoint inhibitors, and immune modulation via therapeutic vaccines or TLR agonists or other approaches discussed here. The challenge will be to define the correct combination and the correct duration of therapy. Given the advances in our scientific understanding of HBV virology and in technologies to translate this knowledge into practical treatments, there is renewed hope that the elusive goal of a cure for chronic HBV may be closer than ever.
